# The Impact of COVID-19 on Postgraduate Training of Doctors in the United Kingdom: A Narrative Review

**DOI:** 10.7759/cureus.33156

**Published:** 2022-12-31

**Authors:** Ahmed Elamin Ahmed

**Affiliations:** 1 Medicine, School of Oncology, The Christie NHS Foundation Trust, Manchester, GBR

**Keywords:** covid-19, trainee well-being, training disruption, uk - united kingdom, pgme postgraduate medical education

## Abstract

The COVID-19 pandemic caused significant disruption to healthcare systems globally. The delivery of medical education was consequently impacted as a result of this. In order to move past the pandemic, we must identify the gaps in postgraduate education. This literature review examines studies focusing on postgraduate training in the United Kingdom (UK) and attempts to bring together the issues that have been highlighted in these studies and the impact that this has had on trainees. It is important for the providers of healthcare education to have an understanding of the impacts of this disruption in order to maintain the quality of postgraduate medical education. Health Education England, along with the Royal Colleges, has published a report that sets a framework on how these issues can be addressed, with some of these changes starting to be implemented in 2022.

## Introduction and background

The arrival of the Coronavirus disease 2019 (COVID-19) to the United Kingdom (UK) in the spring of 2020 led to widespread changes within the National Health Service (NHS) as priorities shifted to tackling the increasing number of cases and treating patients admitted to hospitals. Working hours were adjusted as rosters had to be modified to allow for safe and adequate staffing levels. There was also redeployment of doctors to areas of greater need, such as the intensive care units and emergency departments, away from the department that they were training in. Face-to-face clinic appointments were changed to virtual appointments or cancelled altogether. Rotations to other units and departments, which usually occur every four or six months, were stopped from happening. Junior doctors, defined in the UK as any doctor who is yet to complete their training and become a consultant, were directly affected by many of these changes. Given the number of changes that occurred as a consequence of COVID-19, the impact that these had on postgraduate training needs to be identified, in order for them to be addressed.

Methods

**Figure 1:**
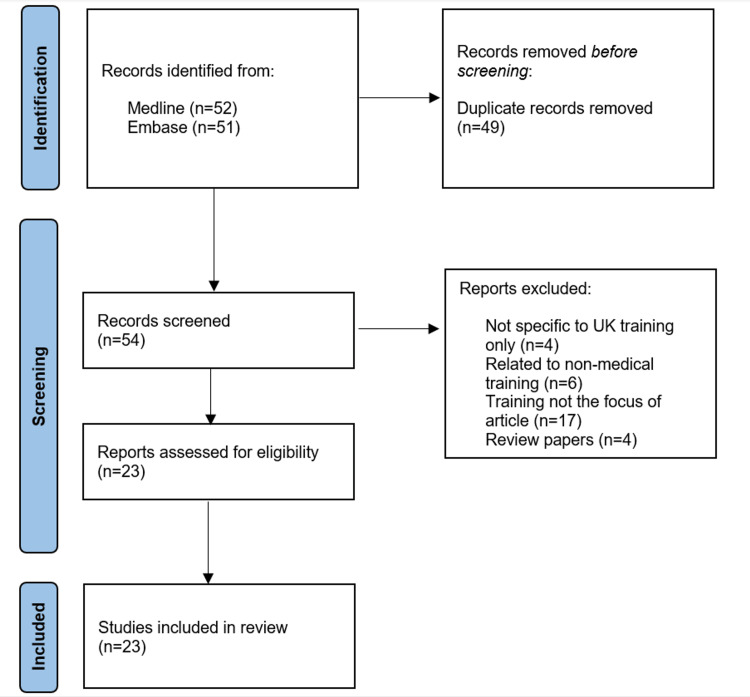
PRISMA flowchart showing the process of study selection PRISMA: Preferred Reporting Items for Systematic Reviews and Meta-analysis.

MEDLINE and Embase were searched up to August 2022 for the terms "Trainee*" or "Medical Education, Graduate" or “Postgraduate education” or “Postgraduate training” AND “United Kingdom” or “UK” or “Great Britain” or “NHS” AND impact or challenge* AND COVID-19. The searches were restricted to published articles, with conference abstracts excluded. Only English language papers were included in the searches. This search resulted in a total of 54 papers after duplicates were removed. Following the title and abstract review, a total of 23 relevant papers were included in the review. The process of study selection is highlighted in a PRISMA flow diagram as shown in Figure 1.

## Review

Of the studies included in the review, 17 discussed the results of surveys completed by trainees and distributed by a number of different organizations [1-18]. Two of the surveys [2,3] were completed by foundation doctors, a doctor who will be in their first two years of working following graduation, within a single region in the UK. The remaining 15 studies [4-18] were national surveys of trainees within a single specialty, facilitated by the training bodies responsible for the training within each of those specialties. These studies produced quantitative data from the answers to the survey questions and qualitative data as they included an option for free text responses, allowing trainees to express specific concerns or impacts that they felt. Three of the studies investigated the changes to services that occurred and the impact that this had on both patients and trainees [19-21]. One study focused on those at the beginning of their training, looking at the impact of the introduction of an "interim" doctor role for medical students who were months away from completing medical school [22]. One study assessed if there had been any change in the technical skills of trainee surgeons, analyzing the results of simulated tasks from before and after the pandemic [23]. The impact of organized formal teaching being changed to a virtual platform was investigated by one study [24]. Surgery was the specialty that had the greatest number of studies (14) on the topic [4,5,8-11,14-19,21,23]. Four were not associated with any particular specialty [2,3,22,24]. The remaining studies focused on the impact of COVID-19 on training in radiology [7,13], pediatrics [12], anesthesia [6], and general practice (GP) [20].

Virtual consultations

In order to increase the number of physicians available to work in in-patient settings, non-urgent clinic services were cancelled. The remaining outpatient services were almost all converted to telemedicine [19]. This not only disrupted the delivery of outpatient services but also resulted in less exposure for trainees to face-to-face consultations This reduced the opportunities available for completing workplace-based assessments as reported in two studies [9,11]. This, however, provided trainees with the opportunity to develop their skills in conducting telehealth consultations, which will be something that they will continue to build on as hospitals look to continue with the model introduced during the pandemic with a mixture of face-to-face and telehealth outpatient appointments [19]. It can also be a cost-effective method of running clinics [25] as it reduces the amount of staff required to work in them and increases the number of consultations that can take place.

GP trainees were also required to convert the majority of their appointments to telemedicine. There was already widespread use of virtual consultations across GP services prior to the pandemic [26], with NHS England planning to increase this [27]. However, the implementation was rapidly accelerated by the pandemic, with the rate of virtual consultations more than doubled during the first three months of the pandemic [20].

Cancellation of non-urgent procedures and investigations

NHS hospitals in England were told to stop all non-urgent elective surgery for three months [28] with guidance from the Federation of Surgical Specialties [29]. The number of studies included in this review highlighted the reduction in trainees gaining operating experience during this time across a broad range of surgical specialties [8,9,11,14-16]. There was a consensus from trainees that the lack of operator experience would require an extension to training time in order to achieve the required competencies [4,12], which was the case for one in eight surgical trainees according to one study [10].

One study looked at quantifying the effect that reduced operating time had on technical surgical skills. The performance of urology trainees on a laparoscopic-simulated assessment was compared between those who completed the assessment before the pandemic and those who took it one year after the start of the pandemic, in 2021 [23]. This showed that the cohort of trainees who completed the assessment in 2021 scored lower results and performed worse than those who did so before the pandemic [23]. However, this study only looked at a small cohort of trainees from a single specialty on a limited number of simulated exercises; therefore, it may not be representative of the wider cohort of trainees, and further work would need to be done to understand the wider impacts that COVID-19 had on the technical skills of trainee surgeons.

A reduction in elective surgeries also resulted in fewer opportunities for anesthetic trainees to improve their skills, with a reported reduction in satisfactory training opportunities for trainees of 65% in 2020 compared to 2019 [30]. According to a survey conducted by the American Society of Anesthesiologists, 23% of trainees were concerned about this drop in the time spent in anesthesia and felt that they would not be as clinically competent as they should be for their stage of training [6]. There is no published data on how many trainees required an extension to their training in order to make up for this. Up to 40% of anesthetic trainees were redeployed to assist their colleagues in the ICU [6], which also would have had an impact on their time in anesthesia. However, it provided an opportunity to learn skills in a similar specialty that will be useful to them in anesthesia.

Surgery and anesthesia were not the only departments that experienced cancellations. One study examined the impact on radiology trainees [7]. This study highlighted that the reduction in radiological procedures resulted in fewer opportunities for trainees to report on computerized tomography (CT) scans, with the proportion of trainees able to report on more than 20 acute CT scans per week falling to 34% from 76% pre-pandemic [7]. This survey was completed in May 2020, only two months after the lockdown had started in the UK. We were unable to infer if these impacts were long-lasting throughout the duration of the pandemic or if this was resolved in order to ensure trainees continued to receive adequate training.

Leadership opportunities

As hospitals were required to make significant changes in a short period of time, there was a requirement for clinicians to assist in facilitating and leading these changes. There were increased opportunities for trainees to develop and improve their leadership and management skills by helping to implement and manage the acute changes within their own work environments. This was highlighted in a survey of pediatric trainees, with 23% of trainees reporting an increase in these leadership and management opportunities [12].

Redeployment

In the spring of 2020, it was anticipated that there was going to be a large peak in hospital admissions of patients with COVID-19. As a result, large-scale workforce changes were required as hospitals sought to increase the number of staff in front-line facing roles managing COVID-19 patients admitted to the hospital. A British Medical Association (BMA) survey [31] conducted in August 2020 showed that 53% of doctors surveyed had been redeployed across the UK. An increased number of doctors was required in the emergency departments, ICUs, and acute medical units. As a result, trainees were moved from their own specialties to cover these gaps [5].

According to the review of the studies, the specialty with the highest proportion of trainees redeployed was radiology, where 76% of trainees were moved to work on the wards [13], followed by oral surgery trainees, where 59% of trainees were redeployed [11]. Due to the cancellation of elective operations, surgical trainees were another cohort that was greatly impacted by redeployment to other areas of need [14,15]. Foundation training doctors were also redeployed to areas with the greatest need [2,3]. Foundation training encompasses the first two years of clinical training, following completion of medical school, prior to starting a specialty training program. These two studies investigated the impact on the foundation trainees within two separate regions of the UK. They highlighted that there were significant differences across geographical areas within the UK for the redeployment of foundation trainees, with 59% of trainees in East England being redeployed compared to 7% of trainees in North Wales. This was likely due to the differences in demand for the areas as the scale of the pandemic varied across different regions. This may be an area of interest in the future if data shows that trainees are progressing at different rates across differing regions.

Trainees had their rotations cancelled in April 2020 on the advice of Health Education England [32], with them resuming again in August 2020. As a result, some trainees would not have gained experience in the specialty into which they were supposed to rotate.

Research

As the pandemic started, all non-COVID-related research was halted, and many centers redirected their efforts to assist with developing research into COVID-19 treatments and vaccines [33]. Research is encouraged throughout the postgraduate training programs, and many trainees take time out of training in order to do so. This redirection of efforts could have resulted in a delay in their work or prevented them from completing their research prior to returning to training.

There were new opportunities for those with limited prior research experience to assist with efforts in investigating the treatment and management of COVID-19. The National Institute for Health Research activated its existing plan for research during a pandemic, and this resulted in 640 research centers being set up in the NHS [34]. This may have been a catalyst for those trainees who were not previously interested in research to continue exploring this further in other areas of interest. One study found that 36% of cardiothoracic trainees were engaged in COVID-19 research in 2020 [4]. This remains to be seen if this will have a lasting impact by increasing the number of clinicians involved in research in the future.

Teaching

At the start of the pandemic, all face-to-face teaching was cancelled across all training specialties to adhere to the guidelines regarding social distancing. There was a rapid implementation of online platforms through which teaching was able to be offered. Organizations were able to offer teaching sessions and webinars to trainees, and this was the most common form of teaching received by these trainees [15]. There are examples of trainees organizing and leading their own virtual teaching programs in order to replace the cancelled face-to-face sessions, with the feedback for one of these programs being very positive [24]. One of the studies highlighted that radiology trainees enjoyed the increased flexibility that virtual teaching afforded them, with 94% of them wanting the new method for the delivery of teaching to remain following the pandemic [13].

Training progression

Trainees are required to have their progress reviewed annually. This ensures that they are achieving the required competencies, allowing them to move onto the next stage of training. They are awarded an Annual Review of Competency Progression (ARCP) outcome following this review of satisfactory or unsatisfactory. Due to the concern that trainees would be unable to attain the requirements to be awarded the ARCP outcome that would allow them to progress to the next part of their training, an alternative option was introduced [35]. These recognized trainees who had been making good progress but had been unable to achieve all the competencies required at that stage due to interruptions from COVID-19. This allowed these trainees to progress to the next stage of their training and helped to review their progress in the following year to see if they had been able to catch up to the expected level. The only exception to this was those who were at a critical progression point of their training, where allowing them to move to the next stage of training without the required competencies may have caused concern for patient safety. They were granted additional training time before progression or completion of the training to allow them to achieve those required competencies. This was the case for 12% of surgical trainees [10] and 5% of obstetrics and gynecology trainees [16].

Changes to the recruitment process had to be made as the delivery of face-to-face interviews was no longer possible. Health Education England, in conjunction with the other corresponding bodies of the UK, issued a statement regarding specialty training applications that training appointments will be made almost entirely on trainee self-assessment scores for those specialties that had not already concluded the interview process and for those starting in August 2020 [36]. This caused concern among trainees applying for higher training, with 69% of anesthetic trainees concerned that these changes would impact them negatively [6]. The following year in 2021, the interview process returned, but this has been delivered via online interviews.

In order to assist with the workforce at the beginning of the pandemic, final-year medical students had the last months of their courses cancelled. They were given the opportunity to join the workforce as “interim foundation doctors” and assist with duties on the front line. A survey of these doctors showed that this actually helped to ease their anxieties about starting work as a doctor [22], and evidence from this may be used in the future to try and help with the transition from medical student to doctor.

Trainee well-being

The pandemic did not only have an impact on the learning experiences of trainees but also negatively impacted their well-being and morale. This has been a common theme that has come out from the surveys that were conducted across all the specialties [4,16,17]. Previous research has already shown that trainees have a higher rate of burnout in comparison to their senior colleagues prior to the pandemic [37]. Burnout of healthcare workers has been shown to have an impact on patient safety and patient satisfaction [38]. A survey of 601 surgeons showed that the prevalence of burnout and adverse mental health outcomes were higher for all trainees when compared to pre-pandemic studies, particularly for those in their first two years of training [18].

Discussion

The articles that have been reviewed highlight the immediate impact that COVID-19 had on postgraduate training in the UK during the first few months of the lockdown in the spring and summer of 2020. The drastic changes that were needed by hospital services to manage the incoming numbers of patients were evidently needed. It was predictable that the required changes would have an impact on the doctors who had to work through that period. One of the unintended consequences of these changes to the healthcare system was the impact on the training of doctors.

The pandemic, however, did not end after this first lockdown in June 2020. The nation endured an extended period of lockdowns until mid-2021, and there continues to be a high burden of COVID within the health service after these ended. Over the last two years, the deliverers of postgraduate education have had time to plan and make adaptations to training programs to mitigate the impacts discussed. More research should be conducted in this area to determine whether there is still an ongoing disruption to training more than two years after the pandemic began so that these issues can be addressed further.

Most of the available literature prior to August 2022 describes the impact that the pandemic had on surgical trainees. This review is limited by the low volume of studies looking at the impact on trainees in other specialties, with no studies looking at the impact on internal medicine trainees, despite them accounting for 31% of all trainees in the UK in 2020 [39]. The steps taken to address the issues raised in these studies may not be applicable to all specialties, and further research is required to investigate the specialty-specific impacts.

This review is also limited by the lack of subsequent research looking at how these changes during the pandemic have impacted the competencies of trainees in the years that followed. Early in the pandemic, trainees reported that they expected it to be more difficult for them to achieve their competencies in the expected timeframe. This is an area that requires further research. This will allow efforts to be focused on areas where there has been a drop in competencies, if any are found.

Most of the evidence used in the literature comes from surveys that were sent to trainees by various organizations. These surveys classically have a low rate of response, as was the case with the majority of these surveys. There is often selection bias within these surveys as those who have had the most extreme experiences are more likely to want to share these experiences and complete the survey.

Planning for the future

Health Education England, along with the other deliverers of postgraduate medical education, acknowledged the impact of COVID-19 on training in April 2021 and signed up to 18 commitments to aid the recovery of training [40]. The modeling they had done had shown that if the recovery of training was not prioritized, the long-term costs to service would be greater. They then followed up with a published report in October 2021 [41], which detailed how they were going to implement this recovery program. Through conversations with each of the Medical Royal Colleges, they looked at how they could address the challenges that had been caused to training due to COVID-19 and designed an overall plan to overcome them. This is a detailed plan aimed at addressing many of the issues that had been highlighted in the literature. The main focus was on reducing the number of trainees who would require an extension to training time; if this was afforded to each trainee, the cost to the healthcare service would be in excess of £350 million [41]. They propose that this can be done by increasing the learning opportunities for trainees through a number of different methods such as allowing trainees to access the independent sector. They have also made a commitment to ensure trainee well-being by continuing to allow flexible working hours and the option to take a break from training if desired.

## Conclusions

While it seems that we are now past the significant disruption to our health systems caused by COVID-19, the impacts on trainees will continue to be felt into the future. As Health Education England's recovery plan was implemented in August 2022, those who were charged with delivering postgraduate medical education have been swift to act to minimize the ongoing impact felt by trainees. It is hoped that the implementation of this plan will help trainees catch up to a point where they will not be at a disadvantage compared to their predecessors who did not have to contend with the disruption of a pandemic to their training. The pace of research into the ongoing effects of COVID-19 on postgraduate education has been slowed down. Understandably, most of the research focuses on the time around the start of the pandemic, when the biggest changes were made. However, this is an area that will need ongoing, continuous work to highlight any persisting issues that are affecting the progress of trainees. Any future research can be used to inform the recovery plans in place.

It is important to note that the disruption had some positive effects on trainees. The dynamic planning that was required created opportunities for leadership and management as trainees looked to take ownership of the decisions affecting their workplaces. The need for research into the disease opened doors to academic career paths that trainees may not have known existed otherwise. Introducing medical students to working in the health service during their final months of study proved an effective way of transitioning from students to doctors, helping to ease anxieties often felt by newly graduated doctors. If a way can be found to ensure that these unexpected benefits can be maintained going forward, alongside the recovery plan in place, then postgraduate medical education may be in a better place than it was prior to the pandemic.
